# Ageing Causes Ultrastructural Modification to Calcium Release Units and Mitochondria in Cardiomyocytes

**DOI:** 10.3390/ijms22168364

**Published:** 2021-08-04

**Authors:** Alessia Di Fonso, Laura Pietrangelo, Laura D’Onofrio, Antonio Michelucci, Simona Boncompagni, Feliciano Protasi

**Affiliations:** 1 CAST, Center for Advanced Studies and Technology, University G. d’Annunzio (Ud’A) of Chieti-Pescara, 66100 Chieti, Italy; alessia.difonso@unich.it (A.D.F.); antonio.michelucci@unich.it (A.M.); simona.boncompagni@unich.it (S.B.); feliciano.protasi@unich.it (F.P.); 2 DMSI, Department of Medicine and Aging Sciences, University G. d’Annunzio (Ud’A) of Chieti-Pescara, 66100 Chieti, Italy; 3 IZSAM, Istituto Zooprofilattico Sperimentale dell’Abruzzo e del Molise G. Caporale of Teramo, 64100 Teramo, Italy; l.donofrio@izs.it; 4 DNICS, Department of Neuroscience, Imaging and Clinical Sciences, University G. d’Annunzio (Ud’A) of Chieti-Pescara, 66100 Chieti, Italy

**Keywords:** excitation contraction (EC) coupling, heart failure (HF), ryanodine receptor (RYR), sarcoplasmic reticulum (SR)

## Abstract

Ageing is associated with an increase in the incidence of heart failure, even if the existence of a real age-related cardiomyopathy remains controversial. Effective contraction and relaxation of cardiomyocytes depend on efficient production of ATP (handled by mitochondria) and on proper Ca^2+^ supply to myofibrils during excitation–contraction (EC) coupling (handled by Ca^2+^ release units, CRUs). Here, we analyzed mitochondria and CRUs in hearts of adult (4 months old) and aged (≥24 months old) mice. Analysis by confocal and electron microscopy (CM and EM, respectively) revealed an age-related loss of proper organization and disposition of both mitochondria and EC coupling units: (a) mitochondria are improperly disposed and often damaged (percentage of severely damaged mitochondria: adults 3.5 ± 1.1%; aged 16.5 ± 3.5%); (b) CRUs that are often misoriented (longitudinal) and/or misplaced from the correct position at the Z line. Immunolabeling with antibodies that mark either the SR or T-tubules indicates that in aged cardiomyocytes the sarcotubular system displays an extensive disarray. This disarray could be in part caused by the decreased expression of Cav-3 and JP-2 detected by western blot (WB), two proteins involved in formation of T-tubules and in docking SR to T-tubules in dyads. By WB analysis, we also detected increased levels of 3-NT in whole hearts homogenates of aged mice, a product of nitration of protein tyrosine residues, recognized as marker of oxidative stress. Finally, a detailed EM analysis of CRUs (formed by association of SR with T-tubules) points to ultrastructural modifications, i.e., a decrease in their frequency (adult: 5.1 ± 0.5; aged: 3.9 ± 0.4 n./50 μm^2^) and size (adult: 362 ± 40 nm; aged: 254 ± 60 nm). The changes in morphology and disposition of mitochondria and CRUs highlighted by our results may underlie an inefficient supply of Ca^2+^ ions and ATP to the contractile elements, and possibly contribute to cardiac dysfunction in ageing.

## 1. Introduction

The cardiovascular system, as many of the others vital systems of the human body, is a target of age-related cellular insults.

Statistics indicate that the risk of heart failure (HF) doubles with each decade of life in individuals aged over 50 [1], making HF the major cause of mortality in the elderlies [2]. However, even if the increased incidence of HF in ageing is undeniable, the existence of a real age-related cardiomyopathy remains controversial [3]. Although in the past years a tremendous effort has been made to unravel the causes of age-related HF [4], the need to fully understand the mechanisms underlying cardiac decline is becoming increasingly urgent as the elderly population continues to grow [5].

The function of striated muscles (skeletal fibers and cardiomyocytes) relies on appropriate supply of ATP and Ca^2+^ ions to the contractile elements. ATP is provided by mitochondria during cellular respiration, and the myocardium is well known to be heavily dependent on the oxidative metabolism [6,7]. Ca^2+^ needed for contraction is released by the sarcoplasmic reticulum (SR) during excitation–contraction (EC) coupling, the mechanism that allows transduction of the action potential of transverse (T)-tubules into release of Ca^2+^ from intracellular stores (the SR). In cardiac cells, EC coupling is based on a mechanism known as Ca^2+^-induced Ca^2+^ release (CICR), where Ca^2+^ entry through Ca.V. 1.2 placed in T-tubules (also known as dihydropyridine receptors, DHPRs) activates ryanodine receptors (RYRs), the SR Ca^2+^ release channels [8].

The intracellular units deputed to EC coupling are named Ca^2+^ release units (CRUs) and are formed by the association of the SR with T-tubules. In skeletal muscle CRUs are also named triads, as they are formed by three elements (a central and narrow T-tubule flanked by two opposed SR terminal cisternae), which are placed at the transition between the I and the A band of the sarcomere. In cardiac muscle cells, CRUs have a different morphology and disposition [9,10]: T-tubules are wider, positioned at the Z line, and are associated to narrower SR terminal cisternae that are wrapped around T-tubules.

The function of mitochondria and CRUs, besides being important for providing independently ATP and Ca^2+^, is also controlled by their cross-communication: indeed, the importance of a close structural association between intracellular Ca^2+^ stores (ER and SR) and mitochondria has been described and discussed extensively in the last couple of decades in both non-excitable and excitable cells [11,12,13,14,15,16,17]. The SR-mitochondria cross-talk in striated tissues has been proposed to be bidirectional [18]: Ca^2+^ released by the CRUs enters mitochondria via the mitochondrial Ca^2+^ uniporter (MCU) to stimulate the respiratory chain, while the reactive oxygen species (ROS) produced by mitochondria can regulate SR Ca^2+^ release [18].

Dysfunction of mitochondria and CRUs has been considered a major determinant of age-related cardiac dysfunction [19,20,21]. One of the first studies proposing a role of mitochondria in age-related dysfunction is dated 1972, when Harman and colleagues [22] hypothesized mitochondria as the main source of ROS. Furthermore, the interplay with the EC coupling system is an important factor to be taken into consideration, as prolonged increases in intracellular Ca^2+^ levels caused by dysfunctional EC coupling may cause excessive Ca^2+^ uptake by mitochondria, which in turn may lead to mitochondrial dysfunction and possibly to death of cardiomyocytes [7,23]. Marks and colleagues proposed mitochondrial Ca^2+^ overload as a key event in HF, as it determines improper ATP production and increased ROS production [24].

Abnormalities in EC coupling have been described in various forms of human HF [25,26,27]. Changes in the organization of the Ca^2+^ release sites due to improper remodeling of the T-tubular network was reported in failing human heart [28]. Non uniform CICR associated to changes in T-tubule organization has been reported in murine, canine, and porcine models [19,20,21]. Other authors had shown that SR Ca^2+^ content, intracellular Ca^2+^ levels, and SR Ca^2+^ release were all reduced in aged cardiomyocytes [29]. Advanced age has been associated with alterations in the expression levels of SR Ca^2+^ ATPases (SERCA) and phospholamban (two proteins involved in SR Ca^2+^ re-uptake), leading to prolonged activation of contractile proteins in cardiac muscles [30]. Finally, calstabin-2, a component of the RYR2 macromolecular complex which modulates SR Ca^2+^ release, was proposed to contribute to age related cardiac alterations [31].

In this work, we have studied the morphology and intracellular disposition of both mitochondria and CRUs in cardiac cells of adult (4 months old) and aged (≥24 months old) mice. Our structural results collected either by confocal and electron microscopy (respectively CM and EM) and our biochemical data, obtained by western blot (WB) analysis, highlight age-related modification of the two machineries. A loss of proper organization of both mitochondria and EC coupling membranes suggests that age-related changes could cause inefficient supply of ATP and Ca^2+^ ions to the contractile elements, and possibly contribute to the cardiac dysfunction.

## 2. Materials and Methods

### 2.1. Animals and Experimental Design

Wild type (WT) C57Bl/6 mice were housed in microisolator cages at 20 °C in a 12 h light/dark cycle and provided free access to standard chow and water. All experiments were performed on cardiomyocytes from WT male mice hearts: papillary muscles were used because of their cardiomyocytes better alignment if compared to those of other regions of the heart. Animals were randomly assigned to two experimental groups: adult (4 months old, *n* = 3) and aged mice (≥24 months old, *n* = 4). All procedures and experiments were conducted according to the National Committee for the protection of animals used for scientific purposes (D. lgs n.26/2014) and were approved by the Italian Ministry of Health (992/2015-PR). Animals were sacrificed by cervical dislocation as approved by the Italian D. lgs n.26/2014.

### 2.2. Electron Microscopy (EM)

WT hearts were fixed by left ventricle injection at room temperature (RT) with 3.5% glutaraldehyde in 0.1 M sodium cacodylate (NaCaCO) buffer (pH 7.4) and then stored in the fixative solution at 4 °C. Papillary muscles were then dissected from whole fixed hearts, post-fixed in 2% OsO_4_ in NaCaCO buffer for 1 h, and en-block stained with uranyl acetate replacement. After dehydration, specimens were embedded in an epoxy resin (Epon 812). Ultrathin sections (~50 nm) were cut using a Leica Ultracut R microtome (Leica Microsystem, Vienna, Austria) with a Diatome diamond knife (Diatome, Biel, Switzerland) and double-stained with uranyl acetate replacement and lead citrate. Sections were viewed in a FP 505 Morgagni Series 268D electron microscope (FEI Company, Brno, Czech Republic), equipped with Megaview III digital camera (Olympus Soft Imaging Solutions, Munster, Germany) and Soft Imaging System at 60 kV.

### 2.3. Immunofluorescence and Confocal Microscopy (CM)

Hearts were fixed by left ventricle injection with 2% paraformaldehyde in phosphate-buffered saline (PBS) for 2 h at RT. Papillary muscles were then dissected, rinsed twice in PBS, incubated for 1 h in PBS containing 1% bovine serum albumin (BSA), 10% goat serum, and 0.5% TRITON X-100 and incubated overnight at 4 °C with appropriate dilution of primary antibody in PBS/BSA. Samples were then rinsed three times in PBS and incubated with a specific fluorochrome-conjugated secondary antibody diluted in PBS solution 1 h at RT and mounted on coverslips with anti-bleach media. Primary antibodies used: mouse anti-RYR2 C3-33 (1:10; Developmental Studies Hybridoma Bank, University of Iowa, IO); rabbit polyclonal anti-TOM20 (1:100; Santa Cruz, Dallas, TX, USA), rabbit polyclonal anti-Junctophilin 2 (y-15) (1:50 Santa Cruz, Dallas, TX, USA); mouse monoclonal anti-caveolin-3 (A-3) (1:50; Santa Cruz, Dallas, TX, USA). Wheat Germ Agglutinin Alexa Fluor 594 Conjugate (W11262; Life Technologies, Waltham, MA, USA) was used to label T tubules. Secondary antibodies used were: Cy3-labeled goat anti-mouse IgG diluted 1:300; Cy3-labeled goat anti-rabbit IgG diluted 1:300; Cy5-labeled goat anti-mouse IgG diluted 1:200, all from Jackson Immuno Research Laboratories (Lexington, KY, USA). Specimens were viewed and imaged using a scanning laser confocal microscope (LSM 510 META Carl Zeiss, Germany) interfaced with an inverted Zeiss Axiovert microscope.

### 2.4. Quantitative Analyses by EM

For all quantitative EM analyses electron micrographs of non-overlapping regions were randomly collected from longitudinal or transversal sections of internal fiber areas taken from cardiomyocytes of adult and aged WT male mice:

(1) Apparently empty cytoplasmic space and mitochondrial volume were determined in electron micrographs from transversal sections using the well-established stereology point-counting technique [32,33] and reported as percentage of the total volume. Briefly, after superimposing an orthogonal array of dots to the electron micrographs, the ratio between numbers of dots falling within mitochondrial profiles and total number of dots covering the whole image was used to calculate the relative fiber volume occupied by mitochondria. In the same way, the ratio between numbers of dots falling within apparently empty cytoplasmic space and total number of dots covering the whole image was used to calculate the relative fiber volume occupied by apparently empty cytoplasmic space. In each specimen, 10 fibers were analyzed, and in each fiber 5 micrographs were taken at 18,000× magnification.
(2) The number of severely altered mitochondria was counted in electron micrographs from transversal sections and reported as percentage of the total number. In each specimen, 10 fibers were analyzed, and in each fiber, 5 micrographs were taken at 18,000× magnification. Mitochondria with any or several of the following ultrastructural alterations were classified as severely altered: (1) mitochondria with clear disruption of the external membrane; (2) severe vacuolization and disruption of the mitochondria internal cristae; (3) mitochondria containing rode-like inclusions, clear matrix and/or lamellar inclusions.
(3) Density of CRUs (i.e., dyads/peripheral couplings) was determined in electron micrographs from longitudinal sections and reported as average number over 50 μm^2^. In each specimen, 10 fibers were analyzed, and in each fiber 5 micrographs were taken at 14,000× magnification. We defined as CRU a T-tubule with associated SR vesicle(s) either single, multiple, or surrounding it.
(4) Size and average number of couplons (i.e., the number of SR elements associated to a single T-tubule) over 50 μm^2^ were determined in electron micrographs from longitudinal sections. In each specimen, 10 fibers were analyzed, and in each fiber 5 micrographs were taken at 56,000× magnification.

For each couplon, we evaluated the following parameters:

(i) Couplons length (i.e., the SR/T-tubule contact length) was measured in random micrographs from WT adult and aged cardiomyocytes. Dyads that did not show clear membrane outlines were not photographed.
(ii) Average area of individual couplons was estimated for each WT sample group (adult vs aged) assuming that EM section cuts across an approximately “circular” junction produces a random cord of such circles. The average measured chord (y) is related to the diameter of the average circle (D) by the equation: y = πD/4. We have used this equation to calculate the average diameter of each couplon and from that the average area.
(iii) The number of RYRs in each couplon was estimated in micrographs from WT adult and aged cardiomyocytes assuming that (a) the couplon is filled with RYR2s, which form ordered arrays touching each other as in [34] and (b) each RYR2 occupies an area of approximately 29 × 29 nm [35].
(iv) The average width of SR cisternae was measured for each WT sample group (adult vs aged) in a high number of SR profile showing clear membrane outline. Three to six lines (depending on the length of the junctional profile) were randomly drawn across the SR and measured.

Morphometric data of couplons size (i.e., couplons length, the estimated size of couplons, the estimated number of RYR/couplons, and the SR width) were obtained using the Analy-SIS software (Soft Imaging System, Munster, Germany) of the EM digital camera (Olympus Soft Imaging Solutions, Munster, Germany).

### 2.5. Western Blot (WB)

Whole intact hearts dissected from euthanized adult and aged mice, were placed in a dish, cut longitudinally in two halves, rinsed in PBS to eliminate excessive blood, homogenized in a lysing buffer containing 3% sodium dodecyl sulphate (SDS) (Sigma-Aldrich, Milan, Italy) and 1 mM EGTA (Sigma-Aldrich, Milan, Italy) using a mechanical homogenizer, and finally centrifuged for 15 min at 900× *g*, at RT. The supernatant was recovered and processed to quantify total protein content. Protein concentration was determined spectrophotometrically using a modified Lowry method, as previously performed [36]. Total protein (20–40 μg) was resolved in 10% polyacrylamide electrophoresis gels, transferred to nitrocellulose membrane, and blocked with 5% non-fat dry milk (Euro Clone, Milan, Italy) in Tris-buffered saline and 0.1% Tween 20 (TBS-T) for 1–1.5 h. Membranes were then probed with primary antibodies diluted in 5% non-fat dry milk in TBS-T overnight, at 4 °C: (a) rabbit polyclonal anti-junctophilin-2 (JP-2) antibody (1:2000; Santa Cruz, Dallas, TX, USA); (b) rabbit polyclonal anti-caveolin-3 (Cav-3) antibody (1:1000; Santa Cruz, Dallas, TX, USA); (c) mouse monoclonal anti-3-nitrotyrosine (3-NT) antibody (1:500; Merck Millipore, Darmstadt, Germany). The mouse monoclonal anti-glyceraldehyde-3-phosphate dehydrogenase (GAPDH) antibody (1:10,000; OriGene, Rockville, MD, USA) was used as a loading control. Membranes were then incubated with horseradish peroxidase-conjugated secondary antibody (1:10,000; Merck Millipore, Darmstadt, Germany), diluted in 5% non-fat dry milk in TBS-T, for 1 h at RT. Proteins were detected by enhanced chemiluminescent liquid (Perkin-Elmer, Groningen, The Netherlands). For 3-NT quantification, we analyzed the whole line for each sample and normalized the average value obtained on the GAPDH signal. Protein quantification was made using ImageJ software (National Institutes of Health, Bethesda, MD, USA).

### 2.6. Statistical Analysis

Statistical analysis was determined using a two-tailed unpaired Student’s *t*-test to compare means of the two groups (adult vs aged), except for data in Figure 3, where significance was evaluated using a Chi-square test. Normal distribution of data in Tables 1 and 2 was checked with the Shapiro–Wilk normality test (GraphPad Prism) and equality of variance was tested with an F test (GraphPad Prism). Statistical analysis in Tables 1 and 2 was determined using a Mann–Whitney U test (GraphPad Prism).

Data are shown as mean ± SEM. Statistical significance was set at *p* < 0.01, or *p* < 0.05, where indicated.

## 3. Results

We analyzed WT hearts from adult (4 months of age) and aged mice (≥24 months of age) using CM, EM, and WB approaches.

### 3.1. Ageing Causes Disarray of Mitochondrial and EC Coupling Systems

**Figure 1:**
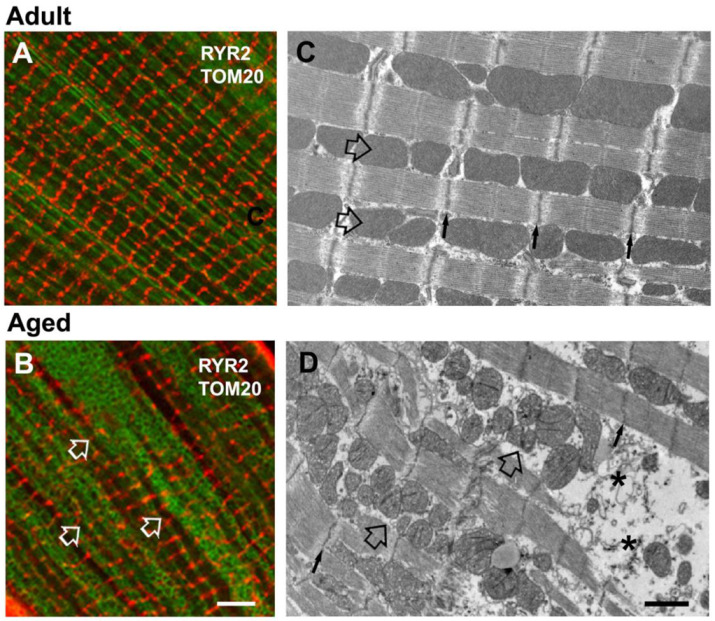
Representative confocal and electron images of cardiomyocytes. (**A**,**B**) CM images of adult and aged cardiomyocytes double-immunolabeled for mitochondria (in green, TOM20), pointed by white empty arrows in B, and RYR2 (in red). See also Supplementary Figure S1. (**C**,**D**) EM images of mitochondria (pointed by black empty arrows) disposed in longitudinal columns between myofibrils in adult and abnormally clustered in aged cardiomyocytes. Asterisks in panel D point to apparently empty cytoplasmic space. Scale bars: (**A**,**B**), 5 μm; (**C**,**D**), 1.0 μm.

**Figure 2:**
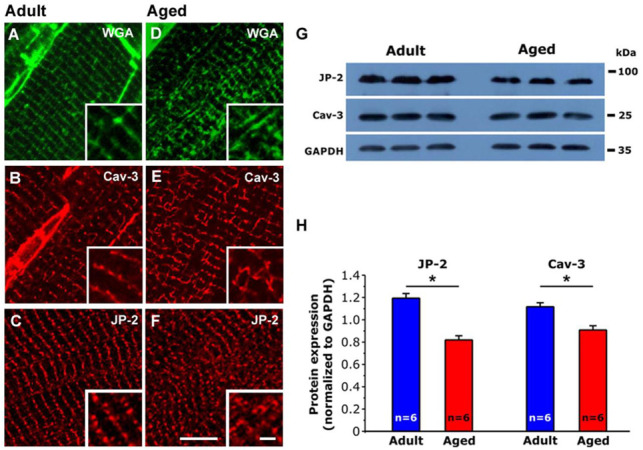
Immunolabeling of T-tubules and WB analysis of junctophilin-2 (JP-2) and caveolin-3 (Cav-3). (**A**–**F**) Immunofluorescence of cardiomyocytes stained with wheat germ agglutinin (WGA) and antibodies against JP-2 and Cav-3. (**G**) Representative immunoblot showing JP-2, Cav-3 and GAPDH (used as loading control), in whole cardiac tissue homogenates from adult and aged mice. (**H**) Bar plots showing the quantitative analysis of the relative JP-2 and Cav-3 expression levels by WB. Data in panel H are shown as mean ± SEM (* *p* < 0.01, unpaired two tailed *t*-test). *n* = number of mice. Scale bar: (**A**–**F**), 10 µm; inset, 2 µm.

We immunostained cardiomyocytes with antibodies marking the position of mitochondria (TOM20) and the position of membrane elements involved in EC coupling, i.e., SR and T-tubules (RYR2 and JP-2 to mark the position of the SR; WGA and antibodies against Cav-3 to mark the position T-tubules) (Figure 1 and Figure 2).

Double-immunolabeling with primary antibodies against RYR2 and TOM20 revealed that in adult cardiomyocytes (a) the EC coupling apparatus has an ordered transversal disposition that creates a cross-striated pattern (Figure 1A; red labeling) while (b) mitochondria are mainly disposed longitudinally between the myofibrils (Figure 1A; green labeling). On the other hand, in ageing cardiac cells the cross-striation generated by the labeling of the EC coupling system is not as precise as in adult cardiac cells (Figure 1B; red labeling) and staining of mitochondria highlighted differences between aged and adult samples (Figure 1B; white empty arrows).

The different immunostaining pattern in aged sample may be the result of the morphological alterations revealed by EM ultrastructural analysis (Figure 1C,D). In adult cardiomyocytes myofibrils are well aligned with one another creating a regular pattern of dark-pale cross-striation (Figure 1C: see small black arrows pointing to Z-lines), while mitochondria are packed in longitudinal columns (Figure 1C; black empty arrows). This organization was compromised in ageing hearts: (a) Z lines were often misaligned (Figure 1D; see small black arrows); (b) apparently empty cytoplasmic space was frequent between myofibrils (Figure 1D; asterisks). In this context of general disarray, also the structure and disposition of mitochondria was compromised, with longitudinal columns being often replaced by formation of abnormal clusters (Figure 1D; empty black arrows).

Data in Figure 1 were also confirmed by images in Figure 2, where we immunolabeled the external membranes (i.e., T-tubule) using WGA (Figure 2A,D) [37] and antibodies against Cav-3 (Figure 2B,E) [38] and JP-2 (Figure 2C,F) [38]. Cav-3 is a protein that plays a key role in T-tubulation, whereas JP-2 is a membrane bridging protein which supports the assembly of junctional membrane complexes by tethering T-tubules to the SR membrane [37,38]: in aged cardiomyocytes the transversal labeling is frequently interrupted by longitudinally oriented fluorescence, indicative of a loos of integrity and organization of the sarcotubular system. By WB we also evaluated the expression levels of either JP-2 or Cav-3 and verified that both were reduced in samples from aged mice (Figure 2G,H), providing a possible molecular mechanism underlying the age-related disarray of the T-tubule network.

**Figure 3:**
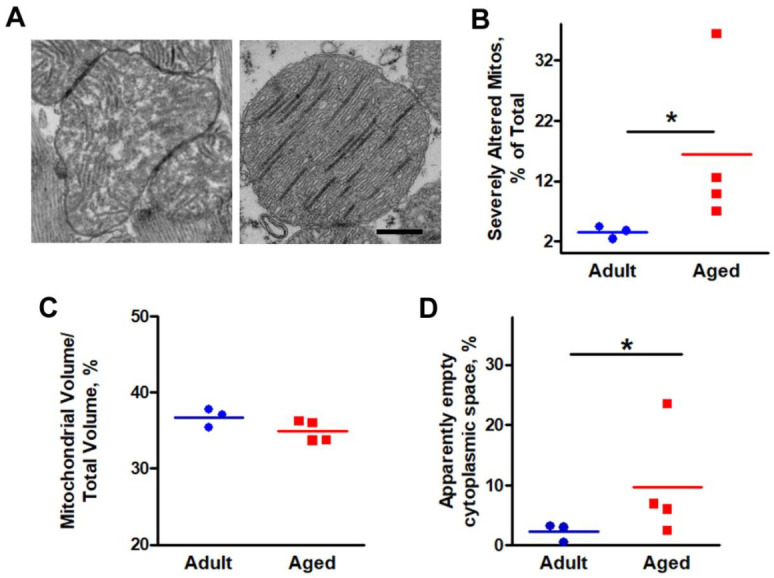
Images and quantitative analysis of mitochondria, and of apparently empty cytoplasmic space. (**A**) Two representative images of severely altered mitochondria. (**B**,**C**) Percentage of severely altered mitochondria and relative fiber volume occupied by mitochondria. (**D**) Percentage of apparently empty cytoplasmic space. See also Supplementary Table S1. (**B**–**D**) Scatter dot plots of individual data for each group are horizontally lined with the mean value (* *p* < 0.01, Chi-square significance test). *n* = 3 Adult mice, 4 Aged mice). Scale bar: 0.5 μm.

In adult cardiomyocytes, mitochondria exhibited an electron dense matrix (Figure 1C) and fairly parallel internal cristae (not shown), while in hearts from aged mice, mitochondria were abnormally shaped, apparently fragmented, and exhibited different type of structural abnormalities. For this reason, in Figure 3, using the well-established stereology point-counting technique [32,33], we performed a quantitative analysis to evaluate the percentage of severely altered mitochondria (Figure 3A,B) as well as the relative fiber volume occupied by either mitochondria and by apparently empty cytoplasmic space (Figure 3C,D).

Whereas the relative volume occupied by mitochondria was unchanged (Figure 3C), ageing caused an increase of both percentage of altered mitochondria (either containing rode-like inclusions, clear matrix and/or damaged internal cristae, vacuoles and lamellar inclusions, etc.) and of apparently empty cytoplasmic space (Figure 3B,D, respectively). See Supplementary Table S1 for the numeric values used in Figure 3B–D.

### 3.2. Measurements of 3-Nitrotyrosine (3-NT) Levels by WB Reveal Increased Oxidative Stress in Cardiomyocytes of Aged Mice

**Figure 4:**
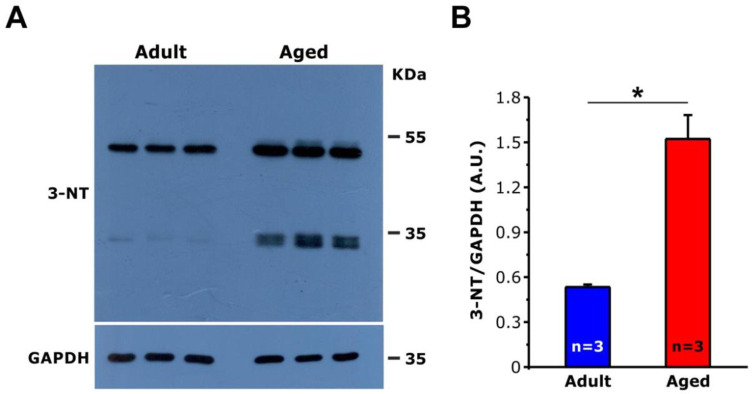
WB analysis of 3-nitrotyrosine (3-NT) for the assessment of oxidative stress. (**A**) Representative immunoblot showing 3-NT and GAPDH (used as loading control), in whole cardiac tissue homogenates from adult and aged mice. (**B**) Bar plots showing the quantitative analysis of the relative 3-NT expression levels by WB, calculated as the ratio between the average value obtained from the signal of the entire lane and GAPDH, for proper normalization. Data are shown as mean ± SEM (* *p* <0.05, unpaired two tailed *t*-test). *n* = number of mice. A.U., arbitrary units.

It is well established that oxidative stress increases with ageing [39]. We also previously reported that oxidative stress is elevated in skeletal muscle from aged mice and may underlie damage to proteins, lipid, nucleic acids, and subcellular organelles, including mitochondria and CRUs [40,41,42]. To evaluate oxidative stress, we measured by WB levels of 3-NT in whole hearts homogenates from adult and aged mice (Figure 4). 3-NT is a product of nitration of protein tyrosine residues mediated by reactive nitrogen species (RNS) such as peroxynitrite anion and nitrogen dioxide, and it is a recognized marker of oxidative stress and oxidative protein alteration [43,44,45,46]. Consistent with the disarray of the T-tubular system and with the increased percentage of severely altered mitochondria in aged compared to adult cardiac muscles, the results from WB experiments showed that levels of 3-NT were markedly augmented in samples of aged mice (Figure 4A), with an average of ~3 times higher than that observed in adult mice (Figure 4B).

### 3.3. EM Analysis Reveals Reduction and Fragmentation of CRUs in Aged Cardiomyocytes

**Figure 5:**
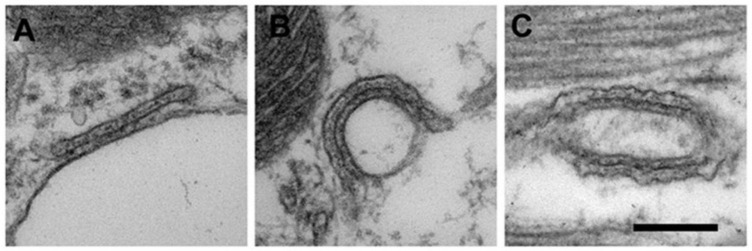
EM images and relative quantitative analysis of CRUs. (**A**–**C**) Representative EM images of a peripheral coupling (PC, panel **A**) and of CRUs formed by either one or two couplons (panels **B**,**C**, respectively). Scale bar: 0.2 µm.

In cardiomyocytes, CRUs are formed by the association of the SR terminal cisternae with either the plasma membrane to form PCs, or with T-tubules to form dyads (Figure 5A,B, respectively). PCs are more frequent during development or maturation, less frequent in adult cardiac cells [8,9]. SR not associated with external membranes (corbular SR), might be also present in cardiomyocytes in which T-tubular network is not well developed, but never encountered in our samples. In adult cardiac cells dyads are for the large majority located in proximity of the Z line, whereas in developing hearts longitudinal dyads (Figure 5C) and dyads at the A band are more frequent [34]. In EM images, the T-tubule may appear associated to a single or to multiple SR elements, forming either one or multiple couplons [34].

Quantitative analysis in Table 1 revealed that, although no significant differences were found in the number of PCs between adult and aged hearts (Table 1, column A), the frequency of internal CRUs decreased significantly with age (Table 1, column B). In addition, the percentage of CRUs located at the A band and of those longitudinally oriented (i.e., not properly placed/oriented) was significantly increased in aged cardiac cells (Table 1, respectively columns C and D), indication of an increasing disorder of membranes forming the EC coupling system.

**Figure 6:**
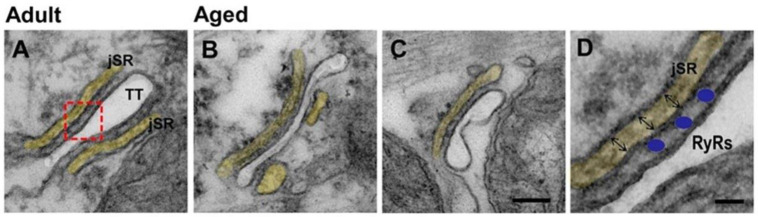
Representative EM images and relative quantitative analyses of couplons. (**A**) A representative CRU in an adult cardiomyocyte formed by two couplons, i.e., a single T-tubule associated with two SR elements. (**B**,**C**) Representative CRUs in aged cardiomyocytes formed by a single T-tubule associated with either a single or multiple SR elements. (**D**) An enlargement of the red box in panel A: the width of the junctional SR (jSR) is shown by small arrows. Legend: SR is false-labeled in yellow; blue dots in panel **D** mark the position of RYR2. Scale bars: (**A**–**C**), 0.1 μm; (**D**), 0.05 μm.

We extended our morphometric analyses of CRUs with the detailed quantitative analyses of couplons presented in Figure 6. In aged cardiomyocytes number of couplons (Figure 6A) was reduced and they appear shorter (Figure 6B,C) than in adult cells. The quantitative analysis in Table 2 confirmed this visual observation: the number of couplons per area was slightly (but significantly) lower in aged cardiomyocytes compared to adult (Table 2, column A), while the average length of individual couplons (a single SR/T-tubule contact) was significantly shorter in aged cardiomyocytes (Table 2, column B).

**Table 1:** Quantitative analysis of PCs, CRUs, and of not properly positioned CRUs (at the A-band or longitudinally oriented).

	A	B	C	D
	No. of PCs/50 μm^2^	No. of CRUs/50 μm^2^	No. of A-Band CRUs/50 μm^2^	N. of Longitudinal CRUs, % of Total
**Adult**	8.7 ± 1.4	5.1 ± 0.5	0.3 ± 0.1	36 ± 10
**Aged**	7.0 ± 1.6	3.9 * ± 0.4	0.7 * ± 0.1	59 * ± 7

Data are shown as mean ± SEM; (* *p* < 0.01, Mann–Whitney U test). *n* = 3 Adult mice, 4 Aged mice.

**Table 2:** Quantitative analyses of number and size of couplons.

	A	B	C	D	E
	No. of Couplons/ 50 μm^2^	Couplons Length (nm)	Estimated Size of Couplons (μm^2^)	Estimated No. of RYR/ Couplons	jSR Width (nm)
**Adult**	7.4 ± 1.2	362 ± 40	0.17	200	26 ± 1
**Aged**	6.9 * ± 1.1	254 * ± 60	0.08	97	32 * ± 2

Data are shown as mean ± SEM (* *p* < 0.01, Mann–Whitney U test). *n* = 3 Adult mice, 4 Aged mice).

We then also estimated (a) the average area of contact between SR and T-tubule in each couplon and (b) the approximate number of RYR2s, which would be contained in a couplon of such size, assuming that the whole area of a couplon is filled with RYR2s (see Methods for additional detail). Couplons in aged cardiomyocytes are significantly smaller in size compared to those of adult and would contain a significantly lower number of RYR2 (Table 2, respectively columns C and D). Finally, we have also measured the average SR width (see small arrows in Figure 6D) and found that it was slightly (but significantly) increased and more variable in shape (as indicated by the higher standard deviation) if compared to that of adult (Table 2, column E). In previous publications we have shown how a wider SR was associated to either lack of CASQ2 or expression of CASQ2 mutants [47,48,49].

## 4. Discussion

### 4.1. Background

The primary function of the heart is to generate the force needed for the generation of blood pressure and its circulation into vessels. To accomplish this primary physiological function of contraction, cardiomyocytes require Ca^2+^, which is finely controlled by CRUs during EC coupling [12], and ATP which is mainly provided by aerobic respiration in mitochondria [50,51]. Mitochondrial function itself is influenced by Ca^2+^ uptake via MCU [52,53].

Increasing age is a main risk factor for cardiovascular diseases and HF, which in turn represent the leading causes of death worldwide [2]. As cellular and molecular modifications occur prior to the functional impairment of the whole heart, understanding the bases of alterations that lead to cardiac cells dysfunction is of central importance to develop therapeutic strategies aiming to prevent cardiovascular and heart disorders.

In the past 10 years, we have investigated age-related changes in CRUs and mitochondria in skeletal muscle fibers [54]. Sedentary ageing causes (a) decrease in the number of sites available for Ca^2+^ release (i.e., the CRUs) [40,41], (b) decreased number and volume of mitochondria [41,42], and (c) alterations in the intracellular disposition and orientation of CRUs and mitochondria with respect to striation of myofibrils [41,42]. These changes are transversal to muscle in mice and human biopsies [40,41,55]. Similar modifications are also caused by short-time denervation [42,56,57,58] but are prevented by regular exercise [42,55].

### 4.2. Main Findings

In this manuscript, we have taken advantage of the previous experience collected studying skeletal [40,41,42,54,55] and cardiac [8,9,10,34,59] muscle to study CRUs and mitochondria and their intracellular disposition, in ageing hearts from mice. The results presented in this manuscript show that ageing in cardiomyocytes causes the following:

(i) loss of architecture and damage of the mitochondrial network: (i) often mitochondria are found abnormally grouped instead of being packed in longitudinal columns (Figure 1); (ii) more frequently mitochondria are swollen and damaged (Figure 3).
(ii) de-modeling of the sarcotubular system: (i) often T-tubules are longitudinal and interrupted causing disarray of the precise cross-striation that characterizes their disposition in adult cardiac cells (Figure 2); (ii) expression levels of JP-2 and Cav-3, two proteins involved in formation and in docking SR to T-tubules in dyads, are reduced, which may contribute to disarray of the EC coupling system;
(iii) increased levels of oxidative stress, measured as 3-NT levels, that in principle could contribute to the damage of organelles (Figure 4); and
(iv) CRUs, the sites of EC coupling, are decreased in number, miss-placed from the correct position at the Z line (Figure 5 and Table 1), and smaller in size (Figure 6 and Table 2).

**Figure 7:**
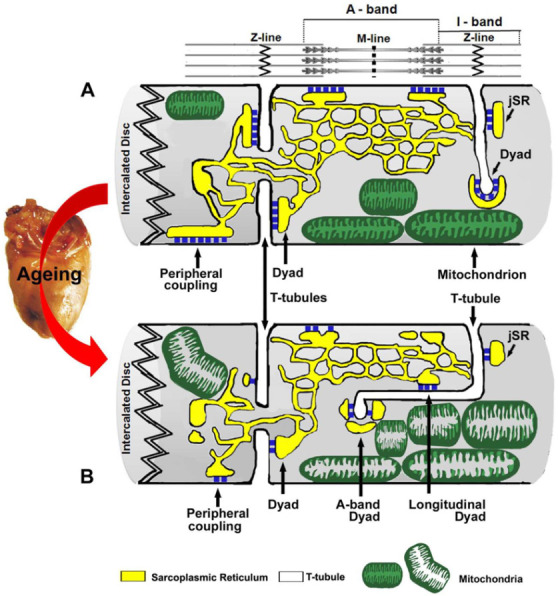
Modeling of age-related changes affecting mitochondria and Ca^2+^ release units (CRUs) in cardiomyocytes. (**A**) Disposition of mitochondria and CRUs in adult cardiomyocyte. (**B**) Aging causes (a) mitochondrial damage (shown in (**B**) by a pale interior) and their abnormal grouping, (b) a significant reduction in the average number and size of CRUs (peripheral couplons and dyads), and (c) an increased frequency of longitudinally oriented dyads. Colour legend: SR in yellow; T-tubule in white; mitochondria in green; RYR2 in blue.

These age-related modifications are summarized by the cartoon of Figure 7. Note that several of these modifications are common to both striated muscle tissues: T-tubules becoming more longitudinal, mitochondrial damage, decrease in number and size and CRUs, etc.

### 4.3. Mitochondrial Network Modifications

The role of mitochondrial dysfunction in the pathophysiology of cardiac ageing has been reviewed by several authors [4,60,61,62,63,64]. Many authors have suggested that the progressive decline in structure, function, and metabolism of cardiac cells is in large part due to an impairment in mitochondrial function and accumulation of mitochondrial DNA (mtDNA) mutations/deletions [60,65,66,67].

Others point to overgeneration of ROS as the central event causing damage to mitochondrial proteins and DNA and organelle dysfunction [22]. The key role of mitochondrial ROS in cardiac ageing is supported by studies where mitochondria-targeted catalase was overexpressed in mice, resulting in improved organelle redox status and increased lifespan [68].

There is general agreement about the fact that excessive Ca^2+^ entry into mitochondria may underlie excessive production of ROS and mitochondrial dysfunction in ageing and disease [24,69]. This Ca^2+^ entry would be mediated by MCU [52,53]: indeed, dysregulation of Ca^2+^ uptake into the mitochondrial matrix has been reported in patients and mice with mutations and knockout of a gatekeeper protein regulating the function of MCU [70,71].

In our aged hearts, we found that oxidative stress is elevated, as 3-NT, a well-recognized and established marker of excessive production of ROS/RNS in the cell, is markedly increased in specimens from aged mice compared to those of adults (Figure 4). Elevated oxidative stress is accompanied by (i) structural damage to mitochondria, which are abnormally-shaped and exhibit different type of structural abnormalities, such as rod-like inclusions; clear matrix; and/or damaged internal cristae, vacuoles, and lamellar inclusions (Figure 3), and (ii) disarray of the general organization of mitochondria and EC coupling systems (see next section for additional detail). In our previous experience studying skeletal muscle, altered mitochondria morphology assessed by EM was often associated to high levels of oxidative stress in models of muscle diseases and ageing [13,41,42,72,73,74].

### 4.4. ECCoupling System Modifications

In cardiomyocytes Ca^2+^ entry from the extracellular space and release by the SR activates contraction of myofibrils. Ca^2+^ entry and release are coupled by a mechanism known as CICR [75]: (i) upon membrane depolarization, a small influx of Ca^2+^ enters through the L-type Ca^2+^ channel (Ca.V. 1.2), also known as DHPR, located mainly in the T-tubule membranes, then (ii) Ca^2+^ entry triggers RYR2 to open allowing release of a larger amount of Ca^2+^ from the SR in the form of discrete release events called Ca^2+^ sparks [76]. In experimental conditions, fusion of sparks result in the propagation of Ca^2+^-waves, a transient rise of cytosolic Ca^2+^ concentration which spreads along the cardiac cells. In physiological conditions, though, the synchronous activation of many sparks induced by depolarization, each of them arising from a different CRU, generates a homogeneous transient that leads to uniform activation of the contractile machinery [77,78,79]. However, the structural disruption of this system in ageing could result in improper activation of the contractile machinery [52,53,54].

Indeed, recent publications have proposed disruption of the systems mediating EC coupling as possible explanation for the impaired cardiac function and loss of contractility [80,81]. In addition, (a) changes in the organization of T-tubules and EC coupling units have been described in failing human heart [28] and (b) improper propagation of CICR associated to T-tubule disorganization has been reported in different animal models of HF [19,20,21]. Although most studies have shown that Ca^2+^ sparks decrease with age, some authors have reported that the expression of both RYR2 channel and DHPR channel do not seem to be affected [82]. However, alterations in the expression levels of proteins involved in EC coupling, such as SERCA and phospholamban, have been also reported in ageing hearts [7].

The results presented in this article show (i) a progressive disarrangement of the T-tubular system (changes similar to those reported following ischemic conditions and cardiac infarction [20,21,22]), and a fragmentation of CRUs in aged cardiomyocytes. These alterations of CRUs include a reduction in number and size of sites of Ca^2+^ release (i.e., couplons), and in partial misplacement of some couplons at the A band from the proper correct position at the Z line (Figure 2, Figure 5 and Figure 6; Table 1 and Table 2). These changes indicate a reduction of sites of Ca^2+^ release and could in principle result in an inefficient CICR propagation [83,84]. In our previous works, we have found similar changes (fragmentation of CRUs), i.e., reduction in size of RYR2 clusters, in mouse models lacking calsequestrin-2 (CASQ2) and carrying human mutations in *CASQ2* linked to catecholaminergic polymorphic ventricular tachycardia [47,48,49].

### 4.5. Closing Remarks

The dysfunction of cardiac cells caused by ageing is responsible for HF. Our results highlight a loss of proper organization and disposition of both mitochondria and EC coupling systems, and fragmentation of CRUs. These results suggest that these age-related changes may cause inefficient supply of ATP and Ca^2+^ ions to the contractile elements, and possibly contribute to cardiac dysfunction in ageing. However, we must underline the limitation of this study, which is mostly based on structural and biochemical analysis, but not yet supported by functional analysis and mechanistic insights. Indeed, molecular changes underlying the loss of proper architecture of these intracellular organelles are unknown and would definitely require additional investigation to be identified. We only reported some changes in expression of proteins involved in SR/T-tubules docking and in T-tubulation (Figure 2), which could affect the proper organization of the EC coupling system, and a significant increase in oxidative stress (Figure 4), which in principle could cause damage to protein, lipid, and DNA and activate the proteolytic machinery.

However, based on our results, we could speculate that the changes we described could interfere with proper CICR, with delivery of Ca^2+^ ions to the contractile machinery, and with CRU–mitochondria signaling. In addition, as the intracellular arrangement of organelles is strictly dependent on the integrity and lateral alignment of contractile elements, we could also speculate that the main cause of the disarray of the mitochondrial and sarcotubular systems could be secondary to the structural disarray of contractile elements, visible as misalignment of Z lines (Figure 1) and as an increased percentage of apparently empty cytoplasmic space (Figure 1 and Figure 3).

## Data Availability

Not available.

## Supplementary Materials

The Supplementary Materials are available online at https://www.mdpi.com/article/10.3390/ijms22168364/s1.

## Abbreviations

3-NT	3-nitrotyrosine
Cav-3	caveolin-3
CICR	Ca^2+^-induced Ca^2+^-release
CM	confocal microscopy
CRU	Ca^2+^ release unit
DHPR	dihydropyridine receptor
EC coupling	excitation-contraction coupling
EM	electron microscopy
HF	heart failure
JP-2	junctophilin-2
RNS	reactive nitrogen species
ROS	reactive oxygen species
RYR	ryanodine receptor
SERCA	SR calcium ATPases
SR	sarcoplasmic reticulum
T-tubule	transverse tubule
WB	western blot
WGA	wheat germ agglutinin
