# Supplementary material for: Ageing Causes Ultrastructural Modification to Calcium Release Units and Mitochondria in Cardiomyocytes
Source: Int J Mol Sci. 2021 Aug 4;22(16):8364. doi: 10.3390/ijms22168364 (PMC8395047; doi:10.3390/ijms22168364)
Supplement: Supplementary file 1 [file ijms-22-08364-s001.zip › ijms-1301456-supplementary.pdf]

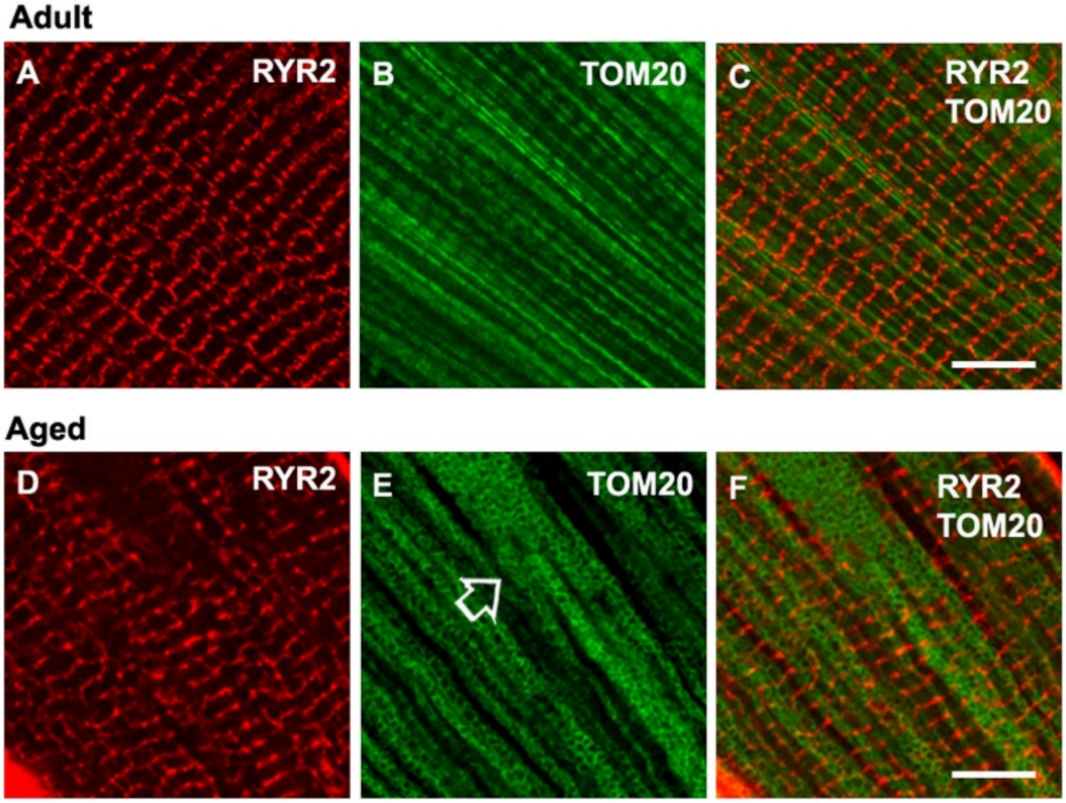

Supplemental Figure S1. Raw image data files of the individual fluorescence channels used to construct the overlays in Figure 1A,B. In these experiments adult and aged cardiomyocytes were double-labeled with antibodies against TOM-20 (in green) and RYR2 (in red). Scale bars: 10 μm.

Supplemental Table S1

	A	B	C
	**Severely altered mitochondria, % of total**	**Mitochondrial vol. / total volume %**	**Apparently empty cytoplasmic space, %**
**Adult**	3.5 ± 1.1	37 ± 1	2.2 ± 0.3
**Aged**	16.5* ± 3.5	35 ± 1	9.7*± 0.6

*Data are shown as mean ± SEM; *p< 0.01 vs adult, Chi-square significance test. Sample Size: 3 adult mice; 30 cardiac cells fibers; 5 micrographs/fiber; 4 aged mice; 40 cardiac cells fibers; 5 micrographs/fiber.*
